# The Mean and the Individual: Integrating Variable-Centered and Person-Centered Analyses of Cognitive Recovery in Patients with Substance Use Disorders

**DOI:** 10.3389/fpsyt.2013.00177

**Published:** 2013-12-24

**Authors:** Marsha E. Bates, Jennifer F. Buckman, Gerald T. Voelbel, David Eddie, Jason Freeman

**Affiliations:** ^1^Rutgers, The State University of New Jersey, Piscataway, NJ, USA; ^2^NYU Steinhardt School of Culture, Education, and Human Development, New York, NY, USA

**Keywords:** cognitive recovery, neuropsychological impairment, longitudinal, person-centered, variable-centered, treatment, substance use disorders, alcohol use disorders

## Abstract

Neuropsychological and cognitive deficits are observed in the majority of persons with alcohol and drug use disorders and may interfere with treatment processes and outcomes. Although, on average, the brain and cognition improve with abstinence or markedly reduced substance use, better understanding of the heterogeneity in the time-course and extent of cognitive recovery at the individual level is useful to promote bench-to-bedside translation and inform clinical decision making. This study integrated a variable-centered and a person-centered approach to characterize diversity in cognitive recovery in 197 patients in treatment for a substance use disorder. We assessed executive function, verbal ability, memory, and complex information processing speed at treatment entry, and then 6, 26, and 52 weeks later. Structural equation modeling was used to define underlying ability constructs and determine the mean level of cognitive changes in the sample while minimizing measurement error and practice effects on specific tests. Individual-level empirical growth plots of latent factor scores were used to explore prototypical trajectories of cognitive change. At the level of the mean, small to medium effect size gains in cognitive abilities were observed over 1 year. At the level of the individual, the mean trajectory of change was also the modal individual recovery trajectory shown by about half the sample. Other prototypical cognitive change trajectories observed in all four cognitive domains included Delayed Gain, Loss of Gain, and Continuous Gain. Together these trajectories encompassed between 86 and 94% of individual growth plots across the four latent abilities. Further research is needed to replicate and predict trajectory membership. Replication of the present findings would have useful implications for targeted treatment planning and the new cognitive interventions being developed to enhance treatment outcomes.

## Introduction

Cognitive impairment is pervasive in persons who enter treatment for alcohol and other drug use disorders (1, 2) and may complicate treatment processes, disrupt interpersonal relationships, and undermine behavioral flexibility and control [e.g., Ref. (3, 4)]. Over the past few decades, tremendous progress has been made in uncovering notable neuroplasticity when substance use stops or is greatly reduced. There is consistent evidence that multiple domains of cognitive functioning, and their underlying brain structures and processes, are capable of recovery (5–7). A recent meta-analysis (8) of the behavioral literature suggested primary cognitive gains occur during the first month or so following cessation of use, with more modest gains over 1 year and even longer term.

A challenge that remains is how to use information about cognitive recovery, or its absence, to guide treatment planning and develop more effective interventions for the cognitively impaired patient who enters treatment for a substance use disorder (SUD). Cognitive recovery in this population has been studied primarily as changes in the mean levels of cognitive and neuropsychological test performance between two points in time. One potential barrier for bench-to-bedside translation is that group summaries may obscure differences between individuals in extent and rate of recovery. The fact that functional relations at the person level cannot be inferred from averaged data has been well articulated in experimental psychology for more than half a century [e.g., Ref. (9, 10)]. The difficulty is not with the averaged data or curves themselves, but rather with the tacit assumption that individual curves, within a range of dispersion, will be of the same form as the average curve (9). A variety of analytic methods are available to empirically evaluate whether this assumption holds in any particular set of data, and if not, whether homogeneous subgroups of persons can be identified that show different and conceptually meaningful patterns of change (9, 11–14). Yet, these methods have not been applied in many areas of behavioral research, leading in some cases to quite erroneous conclusions about typical patterns of change in health status over time. A notable example comes from research on exposure to trauma, where an early focus on psychopathological outcomes and event based comparisons obscured understanding of the typical patterns of response to traumatic (or potentially traumatic) events (15). Bonanno and colleagues used latent growth curve modeling to show that these outcomes could be grouped within prototypical longitudinal patterns of resilience, gradual recovery, delayed increases in distress, and chronic distress, with resilience being the modal trajectory (16). It was the initial studies from this group that used means and standard deviations (SDs) to assess normal variability that established the heterogeneity of outcomes following bereavement and other potentially traumatic events, and set the stage for subsequent research aimed at identifying predictors of resilience [e.g., Ref. (17)].

Similarly, the question arises whether a focus on clinical levels of neuropsychological impairment, or comparisons of cognitive ability in SUD and control samples without SUD, has obscured identification of prototypical trajectories of cognitive change in treatment samples. The ability to identify, and ultimately predict, heterogeneous patterns of cognitive change would have useful implications for clinical decision making, such as the design and pacing of treatment delivery and the need for follow-up assessment and provision of continued care. In this article, we address the inceptive question of whether mean-level improvements observed in different domains of cognitive ability observed in SUD treatment samples represent the modal trajectories of change at the level of the person, or whether there are prototypical longitudinal change patterns that differ substantively in form from the average pattern of change over time. To accomplish this, we integrated what have been termed “variable-centered” and “person-centered” analytic approaches to model mean and person-level cognitive changes over time. Conceptually, variable-centered approaches assume that interrelations among variables reflect how variables function within the person (18). Such approaches, which comprise the predominant statistical methods for predicting outcomes in behavioral research, allow strong hypothesis tests about underlying mechanisms (19, 20). In the context of examining cognitive recovery, the variable-centered approach of structural equation modeling (SEM), and specifically confirmatory factor analysis (CFA), is valuable for the goal of predicting how cognitive constructs (underlying cognitive abilities) influence their indicators (performance scores on neuropsychological tests) [see Ref. (21)]. The significance of changes in underlying cognitive abilities over time can be used to estimate the average level of cognitive recovery following cessation of alcohol and drug use. In comparison, a person-centered approach views functioning and change from a more holistic perspective (13). In longitudinal studies, it is used to characterize change across time within the person and capture intra-individual dynamics (22). The ultimate goal of most person-centered analyses is to group individuals into homogeneous categories or subgroups and to determine the predictors of subgroup membership (21).

The present study used a variable-centered model of cognitive change as a starting point against which to gauge the potential heterogeneity of change patterns observed at the level of individual patients in SUD treatment. We built on our previous CFA of cognitive abilities in a sample of 197 men and women who entered treatment for an alcohol and/or drug use disorder (23–25). CFA was used to specify the degree to which an *a priori* conceptual model^1^ of four underlying cognitive ability constructs supported performance on 15 standardized neuropsychological tests that assessed the major domains of impairment found in heavy, chronic users of alcohol and other drugs (27, 28). We showed evidence of convergent and discriminative validity for underlying ability constructs termed executive function, memory, verbal ability, and complex syntactic information processing speed. Six weeks after treatment entry, participants were again administered the neuropsychological test battery. The four-factor structure observed at baseline was largely invariant (identical factor pattern and, with one exception, identical factor loadings) at the 6-week follow up (24). After accounting for practice effects, as explained below, there were statistically significant mean improvements in the executive, verbal, memory, and complex information processing speed latent constructs across the 6 weeks, consistent with trends in cognitive recovery observed in earlier studies that used different methods [reviewed in Ref. (29)]. The average memory improvement was of medium effect size (ES) (30); improvements in the other three latent constructs were of small ES.

In the present study, we added two additional neuropsychological retests that were conducted in this sample at 26 and 52 weeks post-treatment entry (25) to determine the average level of changes in the cognitive abilities over the full 1-year testing interval. We output latent ability scores for each participant at each of the four test occasions from the variable-centered analysis in order to calculate person-centered empirical growth plots (31–33). Two related aspects of our approach to this problem are noteworthy in addressing practice effects and model invariance.

One challenge of longitudinal studies involving repeated administrations of neuropsychological tests is to determine the relative extent to which changes in test performance over time represent substantive changes in underlying abilities versus the influence of factors such as measurement unreliability or practice with specific tests. Factor loadings represent the degree to which the underlying ability construct supports performance on each test, whereas residual terms [also referred to as “specific factors” (34)] include unique variance of the indicator and measurement error. To the extent that practice effects are relatively test specific, the SEM approach allowed practice effects to be partialed out from “true” improvement in latent abilities. This method of estimating practice effects is an alternative to the use of control groups, offsetting of initial testing, and alterative versions of the tests (35). Although none of these approaches is perfect, the strategy of separating common and specific factors more fully utilizes the strengths of SEM and provides a convergent approach to the more commonly employed methods to control for practice. A related consideration is whether the underlying abilities that supported performance during earlier test administrations are the same as or different from the abilities that were called upon when the tests were performed later (i.e., for the third and fourth time). Tests of invariance of common factor loadings across time were used to examine the extent to which the same underlying latent abilities supported test performance at each testing occasion.

The empirical growth plots of latent construct scores were used to characterize change in cognitive abilities within individuals over the 1-year time span. Change over time can vary in terms of direction, rate, and shape of trajectories as well as in minima and maxima (36). With respect to expected cognitive changes in SUD treatment samples, the previous literature suggests several hypothetical subgroups of growth plots of cognitive change that may be expected to occur with some frequency in the SUD treatment population [e.g., Ref. (35, 37)]. The average trajectory of change over 1 year would be expected to reflect substantive gains in cognitive ability during the initial weeks of abstinence and treatment, followed by continued, yet smaller improvements extending over 1 year (38, 39). Such trajectories would comprise persons showing mild to severe cognitive deficits at treatment entry, primarily related to direct (neurotoxic), and indirect (e.g., medical problems) alcohol and drug effects that would be expected to remediate with abstinence or greatly reduced substance use. This trajectory is consistent with the average group-level improvement observed in previous studies (38, 39). Given that not all individuals show cognitive impairments at treatment entry (40, 41), another prototypical trajectory may reflect average to high stable levels of cognitive abilities over time. Another type of stable trajectory may reflect the consistently lower ability levels of persons who remain impaired across time despite cessation of substance use, including those with more persistent substance-related impairment or premorbid cognitive deficits. In addition, a trajectory indicative of impairment at treatment entry that remediates in the short term, but then reemerges later in time, due to the resumption of substance use or other intervening factors would be expected (42).

Combining variable-centered and person-centered quantitative approaches enabled us to compare the extent to which cognitive change trajectories of individual patients primarily reflected the averaged cognitive changes that were observed in the sample at large, or whether prototypical subgroups of individual-level trajectories emerged that represent alternative patterns of change. The results provide an empirical foundation for future research aimed at identifying predictors of subgroups of individuals who are relatively homogeneous with respect to cognitive recovery and different from persons in other groups (21, 31).

## Materials and Methods

### Participants

One hundred ninety-seven participants (79 women) volunteered to take part in a multi-site addiction treatment study. Treatment sites included two private, hospital-based treatment programs, which offered residential or intensive day treatment (*N* = 119), a day treatment program for older adults with alcohol problems (*N* = 47), and brief interventions offered to patients who screened positive for SUD in an urban medical center (*N* = 31). Exclusion criteria were being <18 years of age (except for the older adult program which had a lower age limit of 60 years), Korsakoff’s syndrome, severe dementia, history of organic brain dysfunction or psychotic disorder, serious medical issues that precluded testing, methadone-maintenance treatment, and an inability to read test materials.

All participants met *Diagnostic and Statistical Manual of Mental Disorders*, third edition, revised [DSM-III-R; (43)] criteria, which was current at the start of data collection, for a current psychoactive SUD. Demographic and substance use characteristics have been previously described in detail (24). In brief, the participants averaged 43.25 (SD = 17.01) years of age, with 12.94 (SD = 2.71) years of education. Sixty five percent identified as Caucasian, 26% as African American, 5% as Hispanic/Latino, and 3% as other. Sixty one percent were employed and 47% married. Medical problems were present in 70.2% of the sample. A sole alcohol use disorder was present in 64% of the sample, a sole drug use disorder in 17%, and the remainder had dual SUD diagnoses. Of those with drug use disorders, cocaine use was reported most commonly, followed by opiate use and marijuana use. The mean full scale Wechsler Adult Intelligence Scale – Revised (44) IQ score estimated from the Shipley Institute of Living Scale (45) was 105.63 (SD = 13.27).

### Measures

Fifteen reliable and valid neuropsychological tests were administered at treatment entry as well as during 6-, 26-, and 52-week follow ups. The test selection rationale and psychometric properties of these tests are described in (23, 24). The specific tests, as well as the sample’s mean performance scores for each test at the four assessment times, are presented in Table 1.

**Table 1:** **Performance on component tests of the neuropsychological assessment battery at treatment entry and at each follow up**.

Neuropsychological test	Treatment entry			6-week follow up			26-week follow up			52-week follow up		
	*N*	*M*	SD	*N*	*M*	SD	*N*	*M*	SD	*N*	*M*	SD
Shipley Institute of Living Scale, Vocabulary^a^	196	27.5	6.6	158	28.1	6.3	119	28.2	6.4	123	28.3	6.6
Shipley Institute of Living Scale, Abstraction^a^	196	20.0	10.1	161	21.6	10.0	143	21.6	10.5	140	22.6	10.2
Word Fluency Test^a^	195	34.7	12.4	162	37.7	13.5	142	37.5	12.3	139	38.2	13.2
Active-Passive Test, affirmative syntax^b^	193	2.7	1.0	158	2.5	0.9	136	2.4	0.9	135	2.3	0.8
Active-Passive Test, negative syntax^b^	193	3.8	1.7	158	3.3	1.4	136	3.3	1.5	135	3.1	1.3
Booklet Category Test^d^*	196	72.3	30.7	155	58.5	31.5	128	53.1	30.7	119	47.4	28.8
Stroop Color-Word Test^a^^,^^c^	162	34.9	12.5	135	39.4	12.3	118	40.1	12.8	115	42.0	13.5
Wisconsin Card Sorting Test^e^	161	5.6	6.2	132	5.0	6.3	95	4.3	5.0	104	3.9	5.0
Digit Symbol Substitution Test^a^	166	47.2	15.7	136	50.4	16.1	120	50.7	16.2	101	53.1	16.4
Trail Making Test, Part A^b^	196	37.6	21.3	162	34.5	18.9	145	33.3	17.9	143	34.3	21.9
Trail Making Test, Part B^b^	195	101.3	72.0	160	91.7	58.4	142	91.7	65.5	141	85.9	59.3
California Verbal Learning Test^a^^,^[Table-fn tfn7]	190	9.0	3.5	158	10.3	3.4	145	10.8	3.6	137	10.9	3.6
Product Recall Test^a^	196	8.0	3.3	161	10.3	2.8	142	10.6	2.8	141	9.9	3.3
Digit Symbol Substitution Test, symbols recalled^a^	166	5.2	2.8	137	6.0	2.8	119	6.1	2.8	116	6.3	2.9
Tower of Hanoi^b^	166	125.0	88.2	136	88.9	66.4	116	78.8	57.7	116	78.6	58.4

^a^Number correct,

^b^time,

^c^participants were screened for color blindness,

^d^errors,

*^e^perseveration errors*.

*The brief version of this test was used (78).

*^
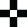
^These data represent a combined mean from the highly correlated long and short delay trials of the CVLT. This mean was then used as a measure of verbal memory in the structural equation model*.

### Procedures

Testing was conducted in compliance with National Institutes of Health guidelines for ethical treatment of human subjects and approved by the Rutgers Institutional Review Board for the Protection of Human Subjects in Research. The neuropsychological assessments were completed using standardized procedures as a component of core assessment battery of a multi-site addiction treatment study. Participants were tested on-site by the same group of interviewers, who were re-evaluated every 6 months to prevent method drift in standardized assessment methods. Participants provided informed consent and were tested approximately 1 week following treatment entry (1 week window), and then were retested 6, 26, and 52 weeks after treatment entry. Each participant was given the same battery of tests that had been administered on treatment entry as well as the Timeline Follow-Back Interview (46). Zero blood alcohol concentration was confirmed at each assessment occasion by means of breath analysis.

### Data analysis

The present results are based on the full sample of 197 participants. Potential bias due to attrition was reduced by estimating model parameters from a full information covariance matrix and maximum likelihood approach with missing data assumed to be missing at random [MAR; (47)] within the Mplus (48) framework. When data are MAR, the variability of SEM-based parameter estimates from sample to sample is less than that obtained when incomplete cases are deleted; to the extent that the MAR assumption is violated, all estimates are biased but those that are SEM-based may be less so (20, 49, 50).

We estimated whether the MAR assumption was valid in this data set. Of a total of 11,820 possible data points with complete follow ups (197 subjects, 4 occasions, 15 cognitive variables), 1.85, 6.57, 10.94, and 10.89% of data points were missing at test times 1, 2, 3, and 4, respectively. Logistic regressions were used to determine for each cognitive test score whether the occurrence of a missing value could be predicted by the subject’s mean score on that variable across non-missing occasions. Of a total of 60 logistic regressions (4 occasions, 15 variables), only 10 were significant at *p* < 0.05. The pattern of results suggested that missing data on two executive function tests (Stroop, Booklet Category) and one memory test (Product Recall) were associated with worse performance on those tests. However, all odds ratios were close to 1 indicating that violations of the MAR assumption were not problematic in this data set.

In the variable-centered stage of analysis, we examined whether the latent structure of cognitive abilities at treatment entry was equivalent across four points in time within the first year after treatment entry, and whether there were significant changes in the means of latent factor abilities over the first year post-treatment entry. Changes in latent means are suggestive of cognitive recovery and changes in the intercepts of manifest test scores suggest practice and other effects that were unique to each of the individual neuropsychological tests.

Our previous CFA at treatment entry yielded a four-factor model (executive functioning, memory, verbal ability, and complex information processing speed) of cognitive abilities that exhibited convergent and discriminant validity (23). In this study, we examined the possibility of systematic differences in cognitive status at treatment entry between participants recruited from the different treatment sites using general linear models performed separately on raw scores from each neuropsychological test. The variance accounted for by recruitment site was in no case significant (all *p*s > 0.05) after variance attributable to age and gender was taken into account (23). We further observed primary factor invariance and significant increases in latent factor ability means at the 6-week follow up (24). In the present study, we extended this CFA to examine factor invariance and changes in factor means at the 26- and 52-week follow ups.

First, to test whether the underlying cognitive construct that supported performance on each of the 15 tests remained stable, the equivalence of the factor loadings (the extent to which performance on an individual test was supported by a given latent ability construct) was assessed across the four assessment occasions. The ability to hold a given factor loading to be invariant across time without causing a significant increment in model misfit (significant increment in chi-square) indicates that the underlying cognitive abilities supporting performance on a given test were stable over time and that the indicators remained reliable indicators of the underlying construct. Second, cognitive recovery at the group level was defined by statistically significant increases in the means of the latent ability factors across time. To help gauge whether statistically significant increases in cognitive performance may be of clinical significance (i.e., likelihood of functional improvements in clients’ ability to learn, retain, or utilize treatment related information), ES measurements were calculated as SDs of change (51). ES differences of 0.2–0.5 were considered small, 0.5–0.8 medium, and 0.8 or greater large. We note however, that this standard has been suggested when considering changes in manifest test scores, which include measurement error; changes in latent construct scores are more sensitive indicators of real change because these scores are not conflated with measurement error and unique measurement effects. Thus, interpretations of clinical significance from standard ESs are conservative.

To account for the influence of repeated administrations of the same neuropsychological tests on performance, the statistical fit of models that held the intercepts of the 15 neuropsychological test scores constant across time was tested. Intercepts of manifest variables would be expected to increase as a result practice effects rather than changes associated with underlying abilities. This interpretation is supported by the consistent lack of evidence in the literature for generalization of training effects across different tests. Intercepts of each manifest test score were initially constrained across all four time-points and significant model misfit was identified by statistically significant modification indices. An inability to constrain a given intercept across two time-points without adversely affecting model fit pointed to the likelihood of performance facilitation due to practice effects or influence of cognitive processes other than the four latent ability constructs represented in the model. Conversely, intercepts that could be constrained without significantly altering model fit suggested that individual test scores co-varied with the means of the underlying latent factors.

The person-centered stage of analysis began by using the best-fitting longitudinal CFA model to estimate four latent factor scores for each participant at each assessment occasion using MPlus software. Next, we used empirical growth plots and temporally sequenced graphs of the latent factor scores (12) to describe each participant’s pattern of change over time. Because the growth plots were generated using latent ability scores output from the variable-centered CFA, it was assumed that they were not confounded with measurement error or unique aspects of individual test performance, and thus more closely reflected true change in underlying ability levels compared to manifest test scores.

Next we explored the prevalence of distinct cognitive change trajectories (prototypical subgroups) by using SDs of the group’s mean-level changes between consecutive assessment occasions (T2–T1; T3–T2; T4–T3). For each of the four latent ability constructs, we classified each participant’s change score as 2, 1, or 0 depending on whether the person’s change score was >1 SD above the mean, within ±1 SD from the mean, or >1 SD below the sample’s mean level of change, respectively. This procedure generated 27 (i.e., 3!) possible change patterns across time. For example, pattern 1 1 1 characterized the change pattern of an individual who did not deviate from the average pattern of change by more than 1 SD over time; the pattern 0 2 1 characterized a delayed pattern of cognitive gains wherein an individual showed less improvement (>1 SD) than the average during treatment, but greater than average improvement (>1 SD) at a later assessment. The 1 SD cut-off was chosen to allow for a reasonable dispersion of trajectories of the same approximate level and form as the average (expected heterogeneity). We then identified alternative change patterns that were exhibited by 5% or more of the sample and categorized trajectories based on commonalities in change patterns. This exploratory method of characterizing prototypical subgroups of change was used in lieu of latent growth mixture modeling or other more powerful technique due to power limitations and the occurrence of partial factorial invariance, as described in Section “Results.”

## Results

### Longitudinal model of latent cognitive abilities over 1 year

**Figure 1:**
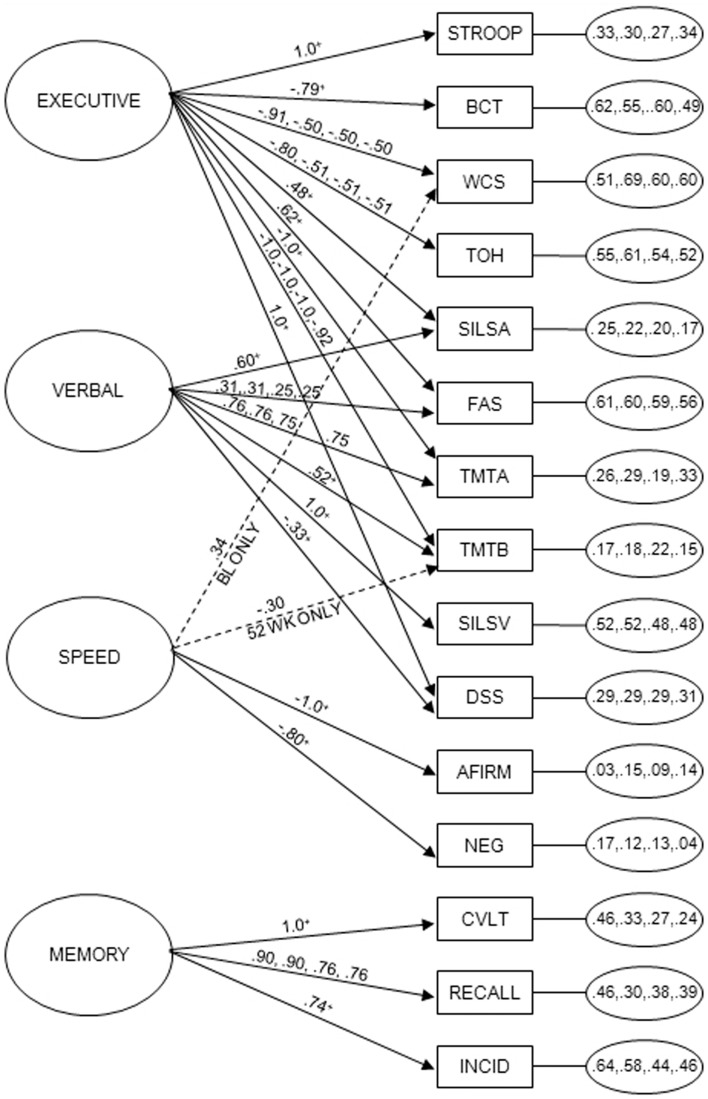
**Partially constrained longitudinal measurement model of 15 neuropsychological tests at four testing times during the first year after treatment entry**. Latent factors, labeled executive, memory, verbal, and complex information processing speed (speed), are located on the left side of the diagram. Residual variances for the indicators at each of the four time-points are in the small ovals on the right side of the diagram. Neuropsychological tests (indicators), shown in squares, included the Stroop Color and Word (STROOP), Booklet Category (BCT), Wisconsin Card Sort (WCS), Tower of Hanoi (TOH), Shipley Institute of Living Scale, Abstraction (SILSA), Word Fluency (FAS), Trail Making Test, Parts A and B (TMTA, TMTB), Shipley Institute of Living Scale, Vocabulary (SILSV), Digit Symbol Substitution, number correct (DSS) and Incidental Memory (INCID), Active-Passive Voice, Affirmative (AFIRM) and Negative (NEG) Syntax, California Verbal Learning (CVLT), Product Recall (RECALL). Arrows extending from latent means to indicators show factor loadings at the four test times. With respect to invariance of factor loadings, of the potential 88 unique loadings (22 loadings over 4 test times), 56 were constrained to identity across 4 test times, 12 were constrained to identity across 3 test times, and 12 were constrained to identity across 2 test times without diminishing model fit. There were no violations of pattern invariance for executive, verbal, and memory factors. For speed factor, there was one unique loading at treatment entry and 1 at 52 weeks. ^+^Loadings constrained across all four test times; ^A^: loadings constrained across 6, 26, and 52 weeks; ^B^loadings constrained across treatment entry and the 6- and 26-week test times; ^C^loadings constrained between treatment entry and the 6-week test time as well as between the 26- and 52-week test times.

The final, partially constrained four factor model is presented in Figure 1. The strength of the loadings of each neuropsychological test on the four latent factors is shown on the arrows extending from the latent factors to the test indicators. The identified model was generally consistent with that previously reported for the first two time-points (23, 24). Although the χ^2^ was significant (χ^2^ = 2494.035, *df*  = 1543, *p* = 0.0000), all other fit indices (RMSEA = 0.056, 90% CI = 0.052–0.060, SRMR = 0.065, CFI = 0.916, TLI = 0.903) suggested an adequate fit of the model to the data (52, 53).

Performance on the neuropsychological tests was, in most cases, supported by the same underlying latent cognitive abilities at each of the four time-points. Partial measurement variance was observed (50). Fifty-six of the 88 factor loadings could be constrained to identity across the four assessment occasions, 12 could be constrained across 3 assessments, and 12 constrained across 2 assessments (Figure 1) without decreasing model fit. There were two violations of factor configural or pattern invariance (54). Specifically, results suggested that the complex information processing speed construct was only a determinant of Wisconsin Card Sorting performance at treatment entry. In contrast, Trail Making Test, Part B performance was more influenced by complex processing speed than by executive function (set shifting, cognitive flexibility) at the fourth measurement occasion. Convergent validity was assessed by testing whether each indicator’s coefficient on its purported latent construct was greater than twice its standard error (55). Validity was supported in 78 of 88 cases; the 10 exceptions represented a weakening of secondary loadings over time, rather than a change in the nature of underlying factors. Discriminant validity was tested by constraining the correlations of factors to 1.0 and performing χ^2^ tests on values from the constrained and unconstrained models (55). All tests were significant, supporting discriminant validity.

### Practice effects

To examine improvements related to repeated exposure to the test materials, the across time invariance of the intercepts of each neuropsychological test was examined. The intercepts of individual neuropsychological tests were initially constrained across all four time-points, and then freed based on model misfit. Ten of the 15 neuropsychological test intercepts could be constrained to identity across all four time-points without diminishing model fit. Five tests showed evidence of practice effects between the baseline and 6-week follow-up assessments: Product Recall, Stroop Color-Word Test, Booklet Category Test, Tower of Hanoi, and Wisconsin Card Sorting Test. The intercepts of Stroop, Tower of Hanoi, and Wisconsin Card Sorting could be constrained to identity across the remaining three test times. For the Booklet Category Test, only the 26- and 52-week assessment intercepts were able to be constrained, indicating that changes in test performance between baseline, 6 and 26 weeks were influenced by repeated test exposure and practice effects. In addition, only the intercepts of the 6- and 26-week assessment of the Product Recall Test were constrained in the best-fitting model, suggesting practice effects were in play between the first two assessments as well as between the third and fourth exposure to the test.

### Mean-level cognitive changes

**Figure 2:**
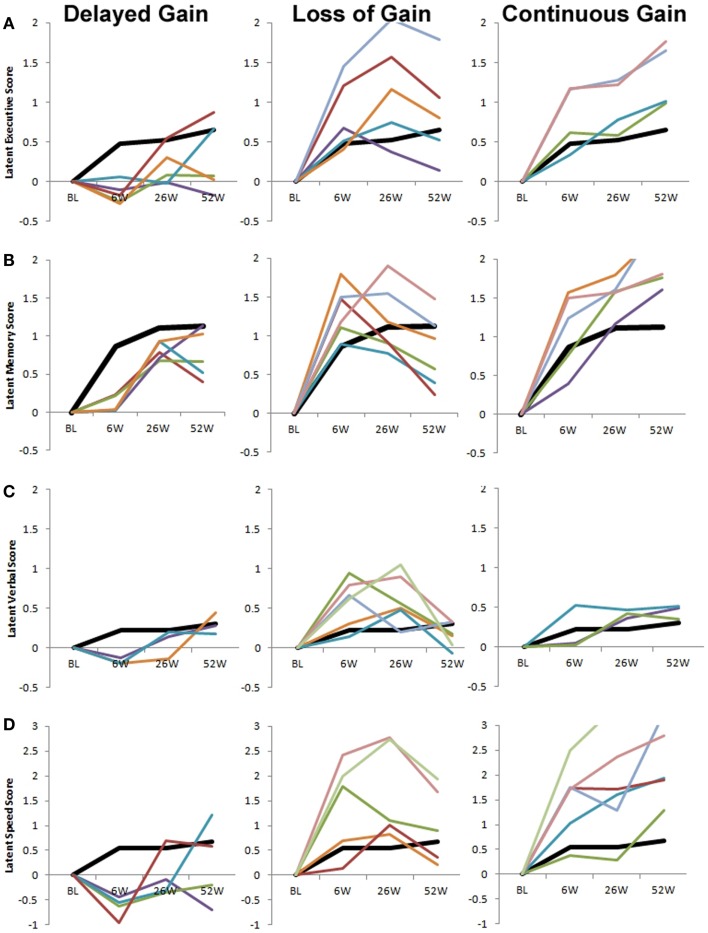
**Prototypical patterns of cognitive change in latent executive (A), memory (B), verbal (C), and complex information processing speed (D)**. In addition to the modal pattern of cognitive change (thick black line in all panels), three consistent patterns of change were observed across all latent factors. The left column depicts trajectories (010, 011, 012, 020, and 021) that show delayed gain. The middle column depicts trajectories (100, 110, 120, 200, 201, 210, and 220) that show early gains that are subsequently partially or fully reversed. The right column depicts trajectories (022, 112, 121, 122, 212, 221, and 222) that show continued gain throughout the first year of recovery. For each panel, data from one individual in each trajectory group are shown. Some trajectories were not observed for each latent factor (see Table 1). To maintain consistency in scale across panels, some trajectory examples include data points outside of the *y*-axis range (e.g., the latent memory and speed, continuous gain group). Note that the scale of latent scores is different for the speed factor **(D)** versus all other factors. BL, baseline/treatment entry; 6W, 6-week follow up; 26W, 26-week follow up; 52W, 52-week follow up.

The majority of gains in the executive (*M* = 0.00, 0.48, 0.53, 0.66), speed (*M* = 0.00, 0.55, 0.54, 0.68), memory (*M* = 0.000, 0.87, 1.11, 1.13), and verbal (*M* = 0.00, 0.23, 0.23, 0.31) cognitive domains was observed between treatment entry and the 6-week follow up, with smaller additional gains over time. Figure 2 (thick black line) shows cognitive recovery at the level of the mean as estimated by comparing the mean scores of the latent factors at sequential time-points in relation to the SD.

### Person-level cognitive changes

The number of participants exhibiting empirical growth plots categorized by the 27 possible cognitive change trajectories is shown in Table 2 for each of the four latent ability constructs. It is noteworthy that the mean change trajectory also was the modal change trajectory (i.e., 1 1 1) comprising 54, 45, 47, and 56% of the individual growth plots of executive, memory, verbal, and complex processing speed, respectively. For the latent executive ability factor, an additional 7% demonstrated a 1 1 0 trajectory (i.e., paralleling the group trajectory from baseline to 26 weeks, but showing less than average change from 26 to 52 weeks) and 6% demonstrated a 1 2 1 trajectory (i.e., paralleling the group trajectory except for a higher than average change from 6 to 26 weeks). No other trajectory group included more than 5% of the sample. For the latent memory factor, 7% demonstrated a 0 1 1 trajectory, indicating a less than average gain during treatment; 5% showed a 1 0 1 and 5% showed a 1 1 0 trajectory suggesting one period of less than average change after treatment completion. Further, 9% showed a 1 1 2 trajectory and 7% showed a 2 1 1 trajectory, indicating one period of greater than average change. For the latent verbal factor, four additional trajectories included >5% of the sample: 0 1 1, 1 0 1, 1 0 2, 2 1 1. For the latent speed factor, one trajectory (1 0 1) contained 6% of the sample; all other trajectories included <5%.

**Table 2:** **Changes in latent factor scores categorized by deviation from group mean**.

	Executive (%)	Memory (%)	Verbal (%)	Syntactic speed (%)	Trajectory group designation
0 0 0	2	1	0	0	*None*
0 0 1	1	0	0	1	*None*
0 0 2	1	0	1	2	*None*
0 1 0	1	2	0	1	Delayed gain
0 1 1	4	7	5	4	Delayed gain
0 1 2	1	1	2	1	Delayed gain
0 2 0	2	2	2	1	Delayed gain
0 2 1	1	4	4	2	Delayed gain
0 2 2	0	1	0	0	*None*
1 0 0	1	2	0	0	Loss of gain
1 0 1	4	5	5	6	*None*
1 0 2	3	0	7	2	*None*
1 1 0	7	5	3	4	Loss of gain
1 1 1	54	45	47	56	Normative gain
1 1 2	5	9	4	3	Continuous gain
1 2 0	1	1	4	4	Loss of gain
1 2 1	6	3	5	4	Continuous gain
1 2 2	0	1	0	0	Continuous gain
2 0 0	0	2	1	0	Loss of gain
2 0 1	0	3	2	1	Loss of gain
2 0 2	1	1	1	2	*None*
2 1 0	1	1	4	2	Loss of gain
2 1 1	4	7	6	4	Continuous gain
2 1 2	3	2	0	1	Continuous gain
2 2 0	1	1	1	1	Loss of gain
2 2 1	0	0	0	2	Continuous gain
2 2 2	0	0	0	1	Continuous gain

*Change trajectories were created by subtracting individual’s latent scores at one test time from his/her scores at the prior test time. Scores were then coded as 0 (>1 SD below the group mean), 1 (within 1 SD of the group mean), or 2 (>1 SD above the group mean) for T2–T1, T3–T2, T4–T3. Trajectories were then grouped based on commonalities in empirical growth curve patterns*.

To identify prototypical *change patterns*, differences in level of ability were removed by subtracting each individual’s latent factor score at treatment entry from his/her latent factor score at each of the test occasions. Each participant’s empirical growth curve then was plotted by trajectory group. In addition to the modal change pattern, three prototypical change patterns were identified across all ability constructs: Delayed Gain, Loss of Gain, and Continuous Gain. Table 2 shows which trajectories were grouped within these prototypical change patterns. Figure 2 shows these prototypical change patterns separately for each of the four latent ability constructs. The thick black line represents the mean (and modal) trajectory of change in each figure. The Delayed Gain groups (Figure 2, left column) include trajectories of less than average improvement during treatment followed by a cognitive gain at the 26- and/or 52-week assessments. Thus, although individual trajectories may have differed in terms of the timing of cognitive gains or losses experienced after treatment, all showed an absence of the increase in cognitive ability most commonly experienced between treatment entry and 6 weeks (i.e., compared to thick black line). The Loss of Gain groups (Figure 2, middle column) include trajectories of average to greater than average cognitive improvement during treatment that is fully or partially reversed later in time. Finally, the Continuous Gain groups (Figure 2, right column) include trajectories of average to greater than average cognitive increases between all test times.

With respect to the hypothesized patterns of cognitive change, the growth plot analyses supported the prototypical trajectory of substantive increases in ability during the first 6 weeks following treatment entry, followed by smaller gains extending over 1 year. Thus, the mean trajectory characterized about half of participants’ individual growth plots. In addition, the expected trajectories demonstrating a loss of gain was found, and accounted for approximately 11–13% of the sample for each ability. In contrast, little evidence was found to support the hypothesized trajectories of stable functioning across time, either at high or low ability levels. Rather, virtually all growth plots represented dynamic changes in underlying abilities over time. Further, unexpected, yet common trajectories (categorized as Delayed Gain) suggested lack of improvement in cognitive ability during treatment, followed by delayed gains in abilities at later assessment times. These trajectories accounted for 8, 15, 12, and 7% of the sample in terms of the latent executive, memory, verbal, and syntactic speed factors, respectively. Finally, 17, 22, 14, and 14% of the sample showed consistent and sustained improvements in function that exceeded expected levels in latent executive, memory, verbal, and complex information processing speed factors, respectively. Overall, the modal and four alternative change groups identified accounted for 90% of the trajectories observed for the latent executive factor, 94% of memory trajectories, 86% of verbal trajectories, and 88% of speed trajectories.

## Discussion

The present study integrated variable-centered and person-centered analytic approaches to capitalize on the unique strengths of each method for understanding of diversity in cognitive changes patterns across a 1-year time span following treatment entry for SUD. The use of CFA to identify underlying cognitive abilities had the advantage of quantitatively examining the fit of an *a priori* conceptual model to the data (56) and its stability over time (57). The person-centered approach captured intra-individual dynamics of cognitive changes that occur at the level of the individual and allowed us to examine the extent to which individual empirical growth plots generally conformed to the average trajectory of cognitive gains observed in the sample as a whole, or could be categorized into representative groups that displayed distinctive patterns of cognitive change. The use of latent ability factor scores in the empirical growth plots decreased the likelihood that the shape of these plots was unduly affected by fluctuations resulting from measurement error and test-specific effects, although caveats related to partial factorial invariance are discussed later in this section.

The group-level analyses revealed average cognitive increases in a sample of adults with alcohol and other drug use disorders between SUD treatment entry and 52 weeks later. The magnitude of the group-level increases across the first 6 weeks suggested recovery of small ES in the domains of executive functioning, complex information processing speed, and verbal ability. The mean increase in memory was of medium ES, suggesting greater likelihood of clinical significance. Some continuing gains were noted across the domains at 26 and 52 weeks, but were relatively smaller in magnitude. Nonetheless, the finding of significant improvement in latent ability constructs at the level of the group is noteworthy, especially in light of the fact that cognitive dysfunction was not an intervention target in the SUD treatments from which participants were recruited and that ES measurements of changes in latent constructs, versus individual test scores, are likely to be conservative.

As previously observed (24), the latent ability factors were highly correlated between successive assessment times, indicating that the rank ordering of individuals within the sample was relatively stable. However, summarizing the bivariate relationships between sequential testing occasions conveys little information about how or in what direction each person changes over time (12). To assess these person-centered changes, we created empirical growth plots for each participant. The plots showed that whereas many participants exhibited cognitive increases that were consistent with changes in the sample’s mean, a sizable number of participants showed trajectories that deviated from the average pattern. Of the 27 possible change trajectories for each latent factor, 21 different trajectories were noted for both the executive and memory factors, 18 for complex processing speed, and 22 for the verbal factor. Thus, not only did a large proportion of participant’s cognitive change trajectories differ from the modal trajectory, the manner in which they differed varied. We were able to qualitatively categorize different trajectories into four prototypical change patterns: modal gain, delayed gain, loss of gain, or continuous gain. The prototypical modal gain trajectory accounted for 45–56% of individual trajectories. Together, the three non-modal change prototypes accounted for an additional 32–49% of the trajectories in latent factor scores. Although these change prototypes did not contain a perfectly homogeneous set of trajectories, they demonstrated commonalities among change trajectories that may be useful for understanding the bi-directional relationship between cognitive abilities and substance use.

Differences between cognitive domains were evident in the individual plots. For verbal ability, the plots showed that for most individuals, the overall pattern of change was very subtle and even changes in the Continuous Gains prototype were not remarkable. This is consistent with the lifespan developmental literature suggesting that crystallized verbal ability tends to increase, or not dramatically decrease, across the lifespan (58, 59), and with the addiction literature showing that crystallized verbal ability is more resistant to alcohol and drug effects than are fluid cognitive skills (60, 61). In the other three cognitive domains, the rate and shape of change within prototypical change groups over 1 year showed more differences across individuals. That some individuals demonstrated lower scores at the 6-week follow-up (immediately after treatment) compared to baseline was unexpected, but may suggest that a subgroup of patients requires additional time to experience the cognitive benefits of sobriety or that contextual factors following treatment may, in some cases, promote cognitive recovery.

The ultimate goal of person-centered analyses is to group individuals into categories, such that each group comprises persons who are similar to each other and different from persons in other groups (21). The description of empirical growth plots provided by the present study provides hypotheses for future studies that integrate variable-centered and person-centered methods to test hypothetical mechanisms that may vary across groups who show differing trajectories of cognitive ability and cognitive recovery. That 6–14% of the sample could not be classified within any of the prototypical change groups points to one of the limitations of the trajectory grouping strategy employed in this study. Although we relied on prospective means and SDs to define trajectories, our use of a 1 SD to define a heterogeneity cut-off was arbitrary in that it imposed pre-defined limits on individual-level data rather that allowing trajectories to emerge (16). The next logical step will be to use the analytic algorithms available to test models that combine class and continuous variables such as latent class growth analysis, latent class cluster analysis, growth mixture modeling, and model-based cluster analysis (48, 62, 63). These methods may prove useful to develop a more personalized approach to cognitive intervention, especially if constellations or patterns of cognitive abilities contribute to behavioral outcomes primarily via the dynamic role they play within the total functioning of the individual [e.g., Ref. (31, 64)].

An important question for future research is whether predictors of different courses of cognitive impairment and recovery can be identified. Our previous study of this sample identified increasing age, less education, and poor medical status as robust and generalized correlates of ability levels at treatment entry, whereas diagnoses of drug use disorders, childhood behavior problems, familial alcoholism, and psychopathology showed small, unique relations to specific latent abilities (23). These risk factors for impairment were generally consistent with those identified in earlier studies (26, 29, 65), and together, explained between 34 and 57% of the true variance in the four latent ability constructs at treatment entry. Predictors of impairment are not necessarily predictors of recovery, and indeed we found this to be the case in explaining gains in abilities that patients had made by 6 weeks (24). Substance use between treatment entry and 6 weeks, and a few additional risk factors were modestly predictive of less recovery, however, they had small, unique ESs. It is possible that because the etiology of cognitive impairment in SUD treatment samples is quite varied (e.g., direct neurotoxic effects, indirect effects mediated by medical disorders and nutrition, traumatic brain injury resulting from increased likelihood of accidental falls, and many others), the predictors of cognitive recovery may also be varied, with individual factors explaining small amounts of unique variance. Likewise, it may be that because patterns of recovery (or absence of recovery) vary, as illustrated by the prototypical change patterns in this study, identifying group-level predictors of cognitive recovery may be difficult in a heterogeneous sample, but improved if subgroups of recovery are identified. Environmental and contextual factors both within and outside of treatment that may promote recovery have received little attention, yet may be important; this is supported by the identification of a prototypical pattern of change that showed little recovery (and, in some cases, further impairment) during treatment but cognitive improvements following treatment.

The approach to generating person-centered trajectories in the present investigation was exploratory do in part to sample size limitations. A larger sample would have allowed for sufficiently sized subsamples who exhibited specific drug use disorders alone or in addition to alcohol use disorders. Drugs such cocaine and opiates have distinct targets within the brain and may have affect cognitive impairment and recovery in differing ways (66, 67). Further, the present sample included persons from three treatment sites. Although this might be considered a strength in improving the ability to generalize results, it was also a limitation in increasing sample heterogeneity. In a larger sample, these sources of heterogeneity could be modeled and invariance across different SUDs and treatment groups could be explicitly tested. In addition, we did not have sufficient power to characterize the influence of different psychoactive medications on impairment. As psychopharmacological treatments for SUD and their common comorbid disorders increase (68, 69), integrated bio-behavioral research is needed to determine their facilitative or disruptive cognitive effects.

Our observations regarding individual trajectories of change are tempered because we did not find full invariance of factor structure over time. Modeling longitudinal changes in behavior assumes that the same construct is measured in the same metric at each assessment time (54). This assumption can be violated whether one is using a manifest scale score or a latent factor score. For example, simply administering the same test on multiple occasions does not insure that the same underlying ability is being assessed, as is often assumed when manifest test scores are analyzed. In a latent variable modeling, such as in the present study, factor invariance, as measured by constraining factor loadings and intercepts to be identical across time is the statistical ideal to ensure that the nature of the construct is not changing across assessment occasions (54). At the same time, these statistical considerations need to be weighed against the conceptual model of the constructs being studied. Thus, intercept invariance over time could not be accommodated in the present model due to practice effects that occur. Note, however, that these practice effects were captured as changes in the intercepts of the specific factors, not changes observed in the latent ability constructs. Whereas there was only one violation of factor loading invariance between treatment entry and 6 weeks in this sample (24), there were additional changes when the longitudinal model extended over 26 and 52 weeks. In the majority of cases, these changes represented a weakening of the latent construct in explaining task performance and were typically of small magnitude. The present results showed, for example, that variance in performance on the Wisconsin Card Sort Test was somewhat less explained by underlying executive functions at later assessment occasions. Such results were not entirely unexpected given the complexity of the present CFA and the occurrence of secondary factor loadings. Future research may benefit from employing less complex characterizations of cognitive abilities to increase the likelihood of measurement invariance over time. Finally, the population studied and the timing of planned assessments was designed to capture a dynamic time span wherein the likelihood of neurocognitive recovery, reorganization, and compensatory processes was high [e.g., Ref. (70)]. Thus, whereas model invariance may be ideal for statistically assessing changes in cognitive ability, identification of factorial variance also could conceivably be relevant for understanding compensatory cognitive processes that support recovery.

The present study did not focus on cognitive impairment defined by clinical cut-off scores *per se*. As expected on the basis of previous studies of addiction treatment samples, however, approximately 70% of the sample was clinically impaired on one or more of the tests at treatment entry (60, 71). Although it still remains to be determined whether cognitive facilitation techniques in addiction treatment should be targeted only to those who have serious levels of cognitive impairment (7), one conclusion from the present results is that almost all persons in the sample showed some extent of cognitive gains during or after treatment. This implies that in the absence of premorbid cognitive ability information, it is likely that most persons enter treatment with reduced ability relative to their own premorbid status, and thus may experience gains due to cessation of substance use, indirect influences such as associated improvements in health, and indirect treatment effects. Results are also consistent with the suggestion that there may be value to SUD treatment development in exploring a broad range of approaches to neurocognitive facilitation and rehabilitation. A resurgence of interest in creating cognitive adjuncts to addiction treatment approaches include techniques to rehabilitate or facilitate cognitive functions by training working memory and other executive and memory functions (72–74), and alternatively, to bolster non-cognitive emotion regulation functions that may indirectly improve cognitive control (25, 75).

Individual growth plot approaches have proved useful in other research areas, but have not been applied to the question of cognitive deficit and recovery during and following addiction treatment. Findings that change processes underlying positive treatment outcomes operated differently in persons with and without executive function impairment (3, 60, 76, 77) suggest that it may be useful to distinguish between individuals who vary systematically in cognitive recovery. This should lead to better understanding the operation of different behavioral change processes within these groups, and to facilitate more effective, personalized treatment approaches.

## Conflict of Interest Statement

The authors declare that the research was conducted in the absence of any commercial or financial relationships that could be construed as a potential conflict of interest.

## Footnotes

^1^Two exceptions were that: (1) performance on visual motor tests was not supported by a unique latent factor but rather covaried strongly with executive performance, as has previously been found (26), and (2) the two syntactic information processing speed measures did not vary with the other verbal ability tests but rather were indicators of a unique complex verbal information processing speed factor.
